# Squamous cell carcinoma of the small intestine: a case report and review of literature

**DOI:** 10.3389/fonc.2025.1550917

**Published:** 2025-04-30

**Authors:** Dandan Wang, Zhe-Xuan Li, Lanlin Hu, Ying Wang, Senlin Xu, Chuan Xu

**Affiliations:** ^1^ Department of Oncology, Affiliated Hospital of Southwest Medical University, Luzhou, Sichuan, China; ^2^ Department of Oncology & Cancer Institute, Sichuan Academy of Medical Sciences, Sichuan Provincial People’s Hospital, University of Electronic Science and Technology of China, Chengdu, Sichuan, China; ^3^ Yu-Yue Pathology Scientific Research Center, Chongqing, China; ^4^ Jinfeng Laboratory, Chongqing, China; ^5^ Department of Pathology, Southwest Hospital, Army Medical University (Third Military Medical University), Chongqing, China

**Keywords:** squamous cell carcinoma, small intestine, clinical feature, treatment, pathogenesis

## Abstract

Malignant tumors derived from the small intestine are rare, and most are adenocarcinomas. Primary squamous cell carcinoma of the small intestine is sporadic with a few cases reported in the literature. This study reports a case of a 56-year-old female who had a history of leakage of exudate for more than 40 years after an appendectomy. The patient presented with increasing leakage of exudate, abdominal pain, and fever this time, and was diagnosed with primary squamous cell carcinoma of the ileal after an intestinal resection and a fascial plasty. The patient declined to receive adjuvant chemotherapeutic treatment and died 9 months after the diagnosis of the tumor. Additionally, we reviewed 26 reported cases, summarized the clinical features and treatments, and discussed the potential pathogenesis and optional therapeutic strategies for primary squamous cell carcinoma of the small intestine.

## 1 Introduction

The incidence of tumors originating from the small intestine (SI) is rare, making up only about 0.6% of all types of cancers and 1-2% of gastrointestinal neoplasms (1, 2). Adenocarcinomas are the primary pathological subtype that accounts for about 30%-50% of small intestinal cancers, followed by carcinoids (28%), leiomyosarcoma (13%), lymphomas (12%), and squamous cell carcinoma (SCC, 0.2%-0.5%) (3–7). The SCC subtype is rare and most of them are metastatic cancers (8–10). To date, the clinical features, pathogenesis, and recommendations of treatment from the Chinese Society of Clinical Oncology (CSCO) and National Comprehensive Cancer Network (NCCN) remain unclear due to the rarity of the primary SCC-SI. The present study reports a case of a 56-year-old female who underwent appendectomy, twice abdominal fistula repair, one intestinal resection due to exudation of incision, and one fascial plasty due to potential inflammation caused exudation of incision. The tissue sample obtained from the initial laparotomy underwent histopathological analysis, leading to a diagnosis of SCC-SI. The patient did not undergo adjuvant chemotherapy. A follow-up telephone call confirmed that the patient passed away due to the tumor in October 2024. In this study, we also reviewed published cases of primary SCC-SI, summarized the clinical characteristics, and discussed the potential pathogenesis, which might provide insight into diagnosis and treatment.

## 2 Case presentation

In January 2024, a 56-year-old woman presented to Emergency Surgery of our hospital with increasing leakage of exudate, abdominal pain, and fever for a month. The patient has a medication history of an appendectomy over 40 years ago followed by two abdominal fistula repairs after three months and 35 years due to the intestinal fistula. No relevant family disease history was identified, and the patient lacked a significant disease history.

**Figure 1:**
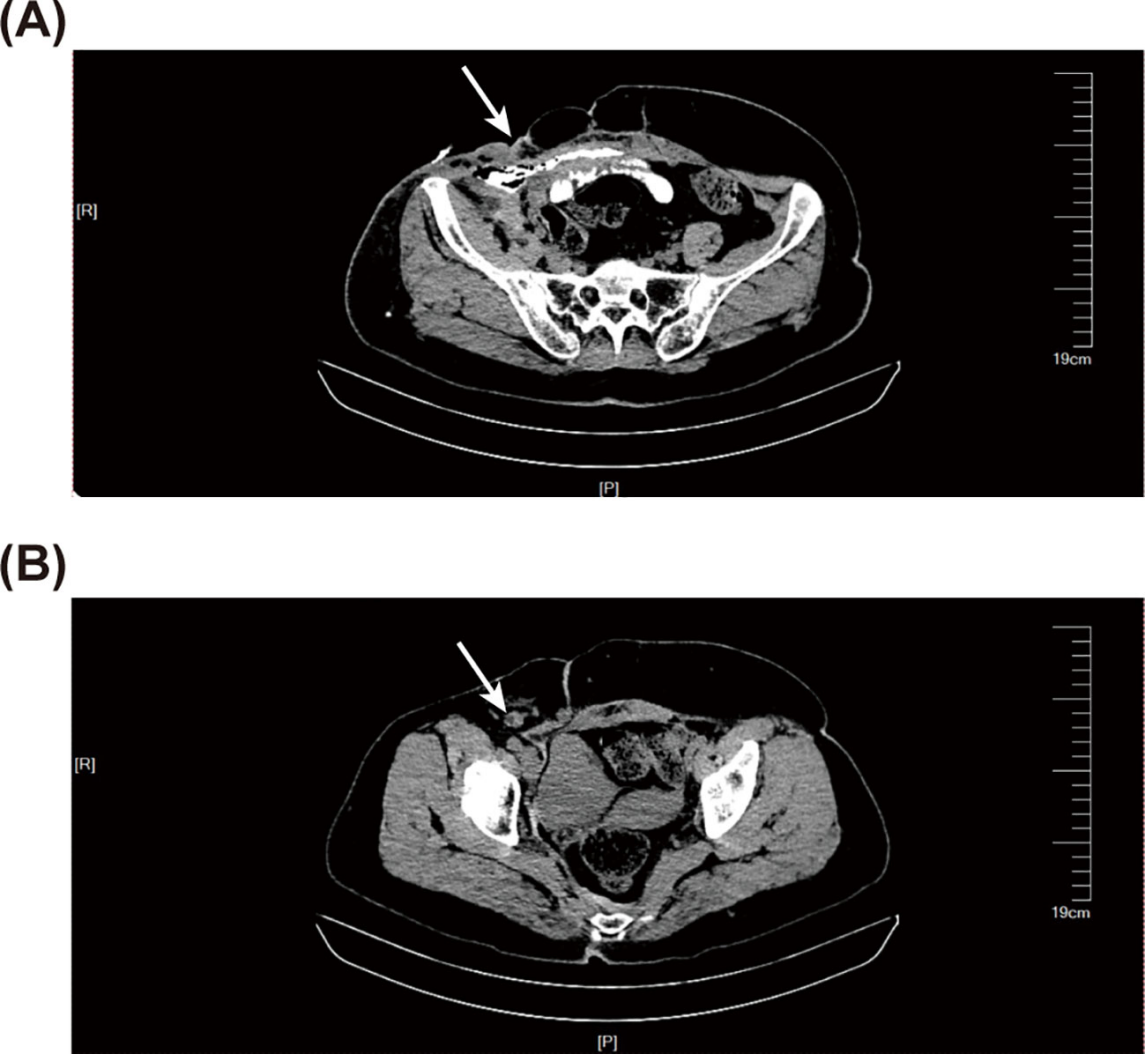
Abdominal computed tomography (CT) before intestinal resection and anastomosis. **(A)** The abdominal CT scan reveals a local sinus tract (the white arrow) in the right lower abdomen, with scattered pneumatosis noted in the fatty layer. **(B)** The abdominal CT scan indicates the presence of enlarged lymph nodes (the white arrow).

During the physical examination, abdominal tenderness was identified in the right lower abdomen, but no peritoneal irritation signs were present. Blood test results indicated that the number of white blood cells was elevated at 13.38 × 10^9^/L (normal 3.50-9.50 × 10^9^/L), the count of neutrophils was 11.380 × 10^9^/L (normal 1.80-6.30 × 10^9^/L), and the level of C-reactive protein was 15.89 mg/L (normal within 5.00 mg/L), suggesting the state of infection. Tumor markers showed a carcinoembryonic antigen of 6.0 ng/ml (normal within 5.0 ng/ml), a ferritin of 358.99 ng/ml (normal within 204.00 ng/ml), and a cancer antigen 125 of 37.60 U/ml (normal within 22.00 U/ml). Abdominal computed tomography (CT) examination displayed a localized sinus tract formation in the right lower abdominal region. Multiple lymph nodes were visible around the sinus tract, some of which were enlarged, with the largest measuring approximately 10mm in diameter. This indicated the possibility of an incisional hernia, coupled with localized infection in the soft tissue and the formation of a sinus tract (**Figure 1**). Abdominal CT imaging revealed no evidence of upper or lower gastrointestinal tract lesions to warrant immediate endoscopic evaluation. Due to the financial burden of additional procedures against the low pretest probability of occult malignancy, the patient declined further endoscopic investigation.

The patient underwent surgical intervention involving resection of a segment of the intestine and subsequent anastomosis on January 16, 2024. In the surgery, an abscess was found on the right abdomen, which was connected to the external environment. Part of the small intestine was embedded within the abscess and showed a two-centimeter rupture with intestinal contents spilling out. However, the patient had an inappropriate dressing change in another hospital, which caused the incision wound infected and leakage of exudate again. Therefore, the patient underwent the second surgery of laparotomy with fascial plasty on April 7, 2024 (**Supplementary Figure 1**).

**Figure 2:**
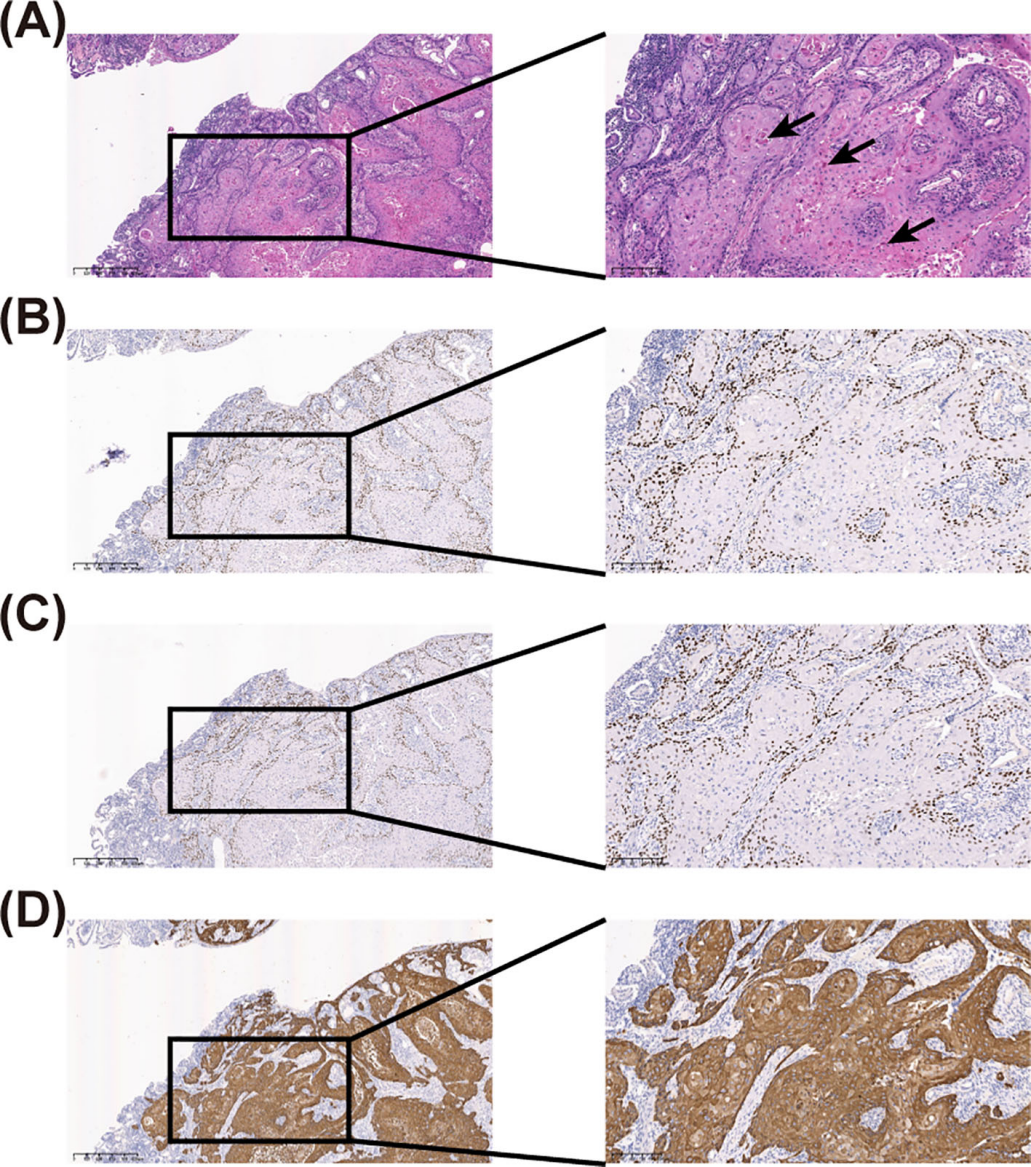
Haematoxylin-eosin (HE) staining and immunohistochemical (IHC) staining of the resected specimen. **(A)** HE staining of the resected specimen, where the black arrow indicates keratin pearls. **(B)** IHC staining shows a positive expression of P40. **(C)** IHC staining shows a positive expression of P63. **(D)** IHC staining shows a positive expression of CK5/6. The left panel is at 40-fold magnification, while the right panel is at 100-fold magnification.

**Figure 3:**
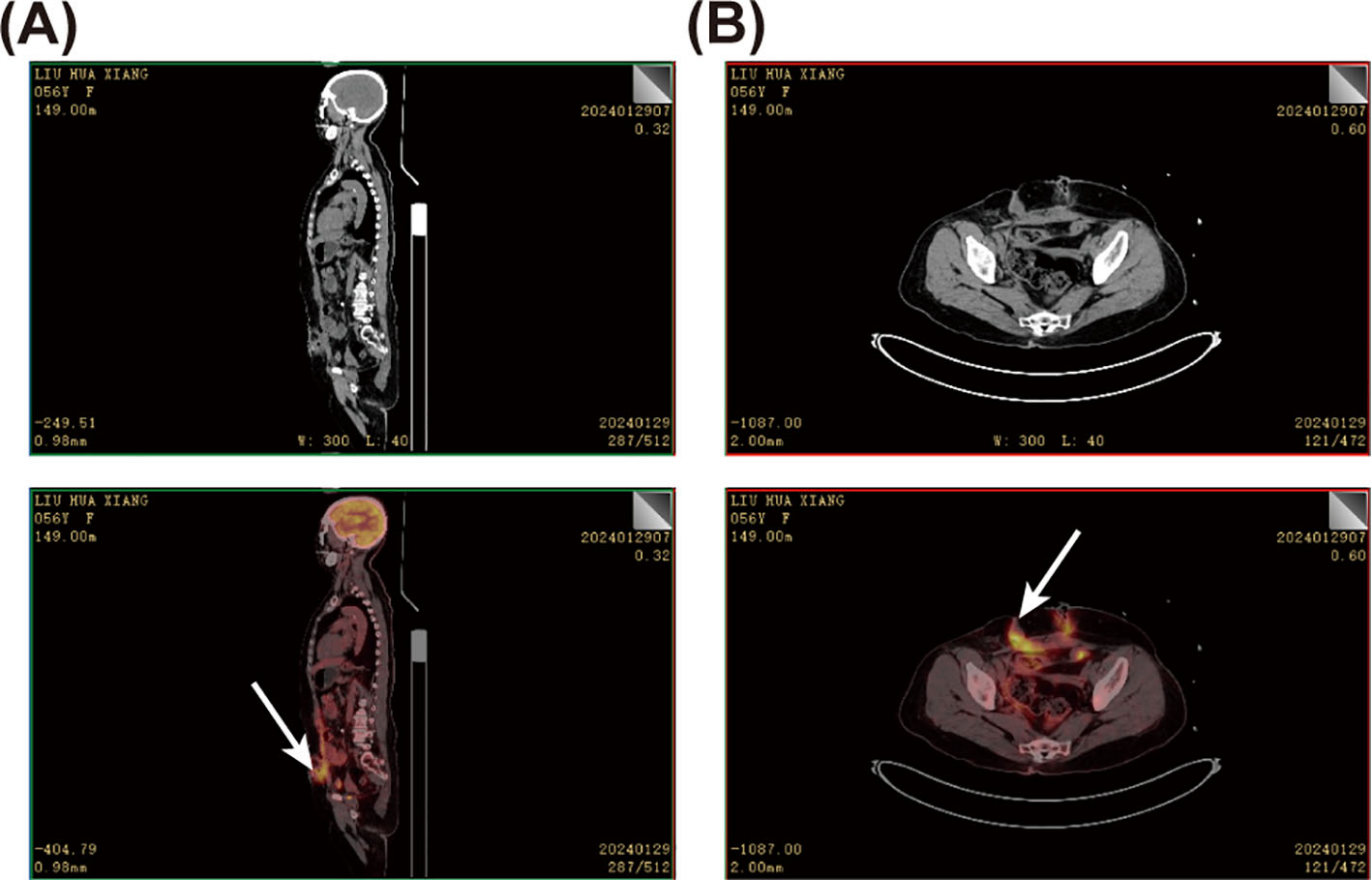
Positron emission tomography-computed tomography (PET-CT) of the patient. **(A)** Sagittal PET-CT image of the patient. **(B)** PET-CT of the abdomen. The white arrow points to the lesion with a high standard uptake value (SUV) uptake, suggesting a high probability of a tumor.

Histopathological examination suggested highly differentiated SCC of the SI, with a maximum tumor diameter of about 4.3 cm, invading the entire intestinal wall. Immunohistochemical analysis reveal that strong cytoplasmic positivity for CK5/6 and nuclear expression of both P40 and P63, markers pathognomonic for squamous differentiation. In contrast, CDX2, a marker typically expressed in intestinal adenocarcinomas, was absent. These findings collectively provide definitive evidence supporting the diagnosis of squamous cell carcinoma (**Figure 2** and **Supplementary Figure 2**). Positron emission tomography/CT did not reveal any tumor lesions in other organs except for the incision area of the right lower abdomen and the middle abdomen (**Figure 3**). This suggests that it is the primary SCC-SI rather than a metastatic lesion. According to the eighth edition of the AJCC staging system for small intestine cancer, the patient is classified as T4N2M0, Stage IIIB. Due to extensive lymph node metastasis in the abdominal cavity, radical resection was considered impossible, and palliative chemotherapy was recommended. However, the patient and his family weighed the side effects and risks of chemotherapy and decided to cease continuous treatment. The most recent follow-up on March 30, 2025, confirmed the patient passed away in October 2024, with an overall survival of 9 months after the diagnosis of the tumor.

## 3 Discussion

The SI, consisting of the duodenum, jejunum, and ileum, measures approximately five to six meters, which comprises 75% of the gastrointestinal tract’s length and accounts for over 90% of its mucosal surface area (11). The malignant tumors originating from the SI are rare, which may be related to reduced mucosal irritation from mechanical or chemical agents regulated by liquidity and alkaline pH, concentrated gastrointestinal lymphoid tissues, low bacteria load, high-leveled benzpyrene hydroxylase, and others (12–17). The small intestinal mucosa is composed of absorptive cells, goblet cells, endocrine cells, Paneth cells, and stem cells but lacks squamous cells (18). Therefore, SCC-SI is infrequent. Additionally, the majority of SCC-SI originates from metastases from tumors in other organs, such as the lung, esophagus, and cervix (19–21). Primary SCC-SI is sporadic and has only been described in some case reports.

**Figure 4:**
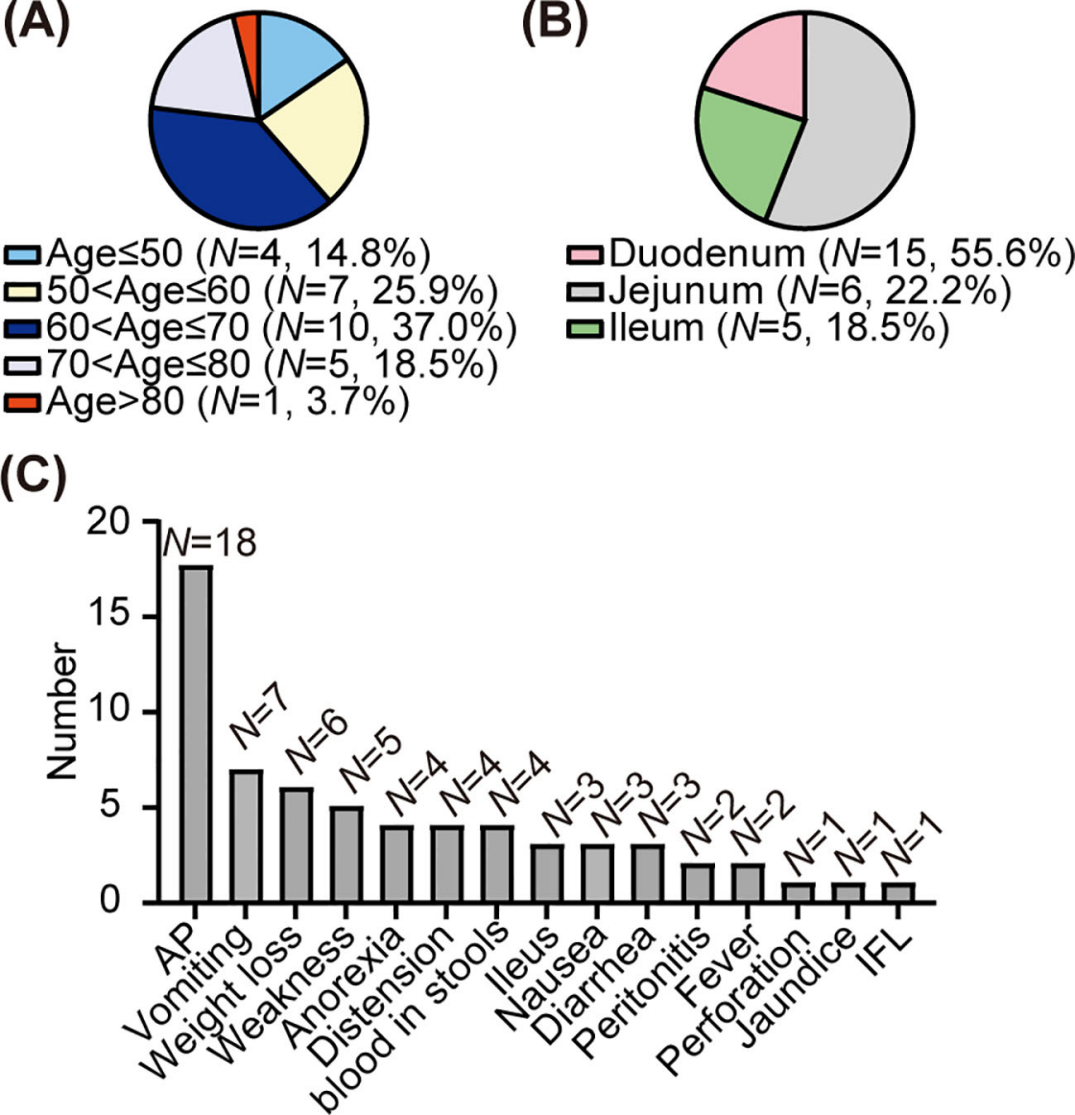
Clinical features of the primary squamous cell carcinoma of the small intestine. **(A)** Age composition ratio among patients with squamous cell carcinoma of the small intestine. **(B)** Composition ratio of lesions in patients with squamous carcinoma of the small intestine. **(C)** The distribution of clinical manifestations among patients with squamous cell carcinoma of the small intestine. AP, abdominal pain; Ileus, intestinal obstruction; IFL, intestinal fistula leakage.

To date, 26 cases of primary SCC-SI (except adenosquamous carcinoma) have been reported in the English literature (**Table 1**) (11, 22–42). Among the one patient in our case and 26 patients in previous cases, the ratio of male to female is 1.5 to 1. The mean and median ages are 63.1 and 65 years (39–91 years), respectively (**Figure 4A**). The lesions were in the duodenum (55.6%, 15/27), jejunum (22.2%, 6/27), and ileum (18.5%, 5/27), with one case each of duplication and diverticula (**Figure 4B**). The symptoms exhibited are abdominal pain (66.7%, 18/27), vomiting (25.9%, 7/27), weight loss (22.2%, 6/27), weakness (18.5%, 5/27), anorexia (14.8%, 4/27), distension (14.8%, 4/27), blood in stools (14.8%, 4/27), intestinal obstruction (11.1%, 3/27), nausea (11.1%, 3/27), diarrhea (11.1%, 3/27), peritonitis (7.4%, 2/27), fever (7.4%, 2/27), perforation (3.7%, 1/27), jaundice (3.7%, 1/27), and intestinal fistula leakage (3.7%, 1/27) (**Figure 4C**). Most patients (77.8%, 21/27) received surgery, while the remaining patients were unable to receive surgery due to extensive tumor metastasis (14.8%, 4/27) or weakness (3.7%, 1/27). For two patients, the treatment plan was not elaborated in detail (29, 39). Eight patients (29.6%, 8/27) received chemotherapy, including four adjuvant chemotherapy after surgery (14.8%, 4/27). Four patients (14.8%, 4/27) received radiotherapy. In previous cases, of the 13 patients with follow-up results, nine patients died when the cases were reported. The mean overall survival for the deceased patients was 13.73 months, with a median overall survival of 5 months. The mean progression-free survival among the four surviving individuals was 26.5 months, with a median progression-free survival of 26 months.

**Table 1:** Clinical characteristics of 27 patients with squamous cell carcinoma of the small intestine.

Gender	Age	Lesion	Symptom	Chronic inflammation history	Stage	Treatment modality	Survival outcomes	Reference
F	59	Small intestine	1. AP 2. Anorexia 3. Ileus 4. Diarrhea	1. Diarrhea for 2 months	^*^T3N0M0, IIA	Sur	Not mentioned	(25)
M	68	Small intestine	1. AP 2. Vomiting 3. Nausea 4. Diarrhea 5. Perforation	No	II	Sur + chemo (Chemo is used after TP: S-1/ED)	PFS: 7 months	(36)
M	58	Duodenum	1. Vomiting 2. Nausea	No	T3N1M0, IIIA	Sur + chemo (5-FU/CIS)	Not mentioned	(45)
M	45	Duodenum	1. AP 2. Weight loss 3. Blood in stools 4. Fever	No	Not mentioned	Sur + chemo (CAPOX)	Not mentioned	(41)
F	75	The first duodenum	1. Blood in stools	No	^#^T4N0M0, IIB	No	Not mentioned	(29)
F	47	The second duodenum	1. AP 2. Weight loss 3. Blood in stools	No	T4N0Mx, IIB	Sur	PFS: 6 months	(37)
M	75	The second duodenum	1. Vomiting 2. Weakness	No	Not mentioned	Chemo + radio	OS: 17 months	(31)
F	58	The second duodenum	1. AP	No	Not mentioned	Chemo + radio	OS: 21 months	(31)
M	54	The second duodenum	1. AP	No	Not mentioned	Sur	Not mentioned	(31)
M	75	The second duodenum	1. Vomiting 2. Weakness 3. Nausea	No	Not mentioned	Chemo + radio	OS: 17 months	(30)
M	65	The third duodenum	1. AP 2. Weakness	1. CD 2. AS 3. OA	T4N0Mx, IIB	Sur	OS: >60 months	(33)
M	61	The third duodenum	1. Weight loss 2. Distension	No	^*^TxN1M0, IIIA	Sur	PFS: 16 months	(26)
F	60	The third duodenum	1. AP	No	^#^T4N2M0, IIIB	Chemo	OS: 1 month	(34)
F	68	The third duodenum	1. Distension	No	Not mentioned	Sur	Not mentioned	(40)
M	39	Diverticula in the third duodenum	1. AP 2. Vomiting 3. Weakness 4. Anorexia	No	^*^TxN0M0	Sur	PFS: 10 months	(22)
M	49	The third duodenum to the proximal jejunum	1. Weight loss 2. Anorexia 3. Distension	No	Not mentioned	No	Not mentioned	(39)
F	55	Jejunum	1. AP	No	^*^T3N0M0, IIA	Sur	Not mentioned	(42)
M	65	Jejunum	1. AP 2. Vomiting 3. Weight loss 4. Distension 5. Diarrhea	No	^*^T3N0M0, IIA	Sur + radio	Not mentioned	(23)
M	80	Jejunum	1. AP 2. Peritonitis	No	^*^T3N0M0, IIA	Sur	OS: 0.8 month	(35)
M	69	Jejunum	1. AP 2. Peritonitis	1. Diabetes	^*^TxNxM1, IV	Sur	OS: 1 month	(11)
M	91	Jejunum	1. AP	No	Not mentioned	Sur	OS: 0.8 month	(38)
F	72	Ileum	1. Ileus	No	^*^T4N0M0, IIB	Sur + Chemo (CARB/PAX)	PFS: 55 months	(32)
F	56	Ileum	1. AP 2. Fever 3. IFL	40-year history of post-surgical fistulas/chronic inflammation	T4N2M0, IIIB	Sur	OS: 9 months	–
F	65	Distal ileum	1. AP 2. Distension 3. Blood in stools 4. Ileus	No	^#^T3N0M0, IIA	Sur	Not mentioned	(24)
F	65	Distal ileum	1. AP	No	^*^T4N0M0, IIB	Sur	PFS: >48 months	(28)
M	62	Distal ileum	1. AP 2. Vomiting	No	^*^T4N1M0, IIIA	Sur	PFS: >36 months	(27)
M	68	Ampulla of Vater	1. Weight loss 2. Weakness 3. Anorexia 4. Jaundice	No	T3N0M0, IIA	Sur	PFS: 4 months OS: 5 months	(51)

*At least; #Approximately.

PFS (Progression-free survival) is defined as the time from the initial diagnosis to tumor progression or the last follow-up time described in the reported cases. OS (Overall survival) is defined as the time from the initial diagnosis to the patient’s death due to any cause.

Sur, Surgery; Chemo, Chemotherapy; Radio, Radiotherpy; AP, Abdominal pain; Ileus, Intestinal obstruction; IFL, Intestinal fistula leakage; TP, Tumor progression; CD, Chronic dyspepsia; AS, Arteriosclerosis; OA, Osteoarthritis; S-1, Tegafur Gimeracil Oteracil Potassium Capsule; ED, Endostatin; 5-FU, 5-Fluorouracil; CARB, Carboplatin; CIS, Cisplatin; CAPOX, Oxaliplatin and capecitabine; PAX, Paclitaxel.

SCC-SI is rare and has a poor prognosis, with its underlying pathogenesis remaining unclear. Existing literature has reported three possible pathogenesis. First, the pluripotential stem cells develop into squamous cells, which subsequently undergo a malignant transformation, becoming cancer cells. In the case reported by Barnhill et al. duodenal carcinoma encompasses three components: adenocarcinoma, neuroendocrine carcinoma, and SCC, suggesting the potential for trilineage differentiation of stem cells (43). However, the detailed mechanism that prompts pluripotent stem cells to differentiate into squamous cells remains unknown. Second, the ectopic squamous epithelium undergoes malignant transformation. Cases of SCC-SI originating from duplication and diverticula have been reported (22, 23). Intestinal duplication and diverticula in the SI are predominantly congenital structural abnormalities. The lining of these structures consists of a mucous membrane that can be similar to the membrane of the adjacent hollow viscus. Still, in some cases, it may display a higher degree of heterotopy or be of a more primitive type (23). These congenital abnormal structures can undergo malignant transformation leading to tumors, although the stimulatory factors remain unclear. Third, the original glandular epithelium of the intestine undergoes squamous metaplasia under stimulation, followed by malignant transformation. This is supported by the observation of multiple foci of squamous metaplasia in the glandular epithelium adjacent to the tumor in some cases (24, 25). Dockerty et al. proposed that continual damaging effects cause glandular epithelium to be replaced by basal or reserve cells, ultimately leading to anaplasia in the basal cells (32). In the SCC-SI, the stimulation factor of squamous metaplasia may be inflammation. In the case described by Friedman, the mucosa adjacent to the tumor tissue showed pronounced chronic inflammation with visible squamous metaplasia (26). In this case, the medical history of more than 40-year intestinal fistula leakage indicated chronic inflammation in the local tissues. The elevated inflammatory indicators, the soft tissue infection revealed by abdominal CT, and the presence of abscesses observed during the surgery support the existence of inflammation. Additionally, the histopathological examination of the excised part of the small intestine embedded within the fistula identified SCC. Our case may support the pathogenesis by which inflammation leads to squamous metaplasia of the local intestinal mucosal glandular epithelium. However, the patient, in this case, also had a medical history of an appendectomy and two abdominal fistula repairs before the diagnosis of SCC-SI. The incisions for all three surgeries were close to the fistula and the SCC-SI. Previous studies have found that residual implantation of squamous cell epithelial cells due to trauma and surgery is one of the pathogenesis of primary SCC of the colon (44). The small intestine is anatomically adjacent to the colon. Therefore, we speculate that surgeries in this patient may have led to the implantation of squamous epithelial cells in the small intestine, which may have facilitated the progression of SCC-SI.

There is no definitive therapy for primary SCC-SI. According to reported cases, surgical resection was prioritized due to localized abdominal disease and symptomatic relief, despite extensive lymph node metastasis. Palliative chemotherapy (carboplatin/paclitaxel) was recommended based on efficacy in metastatic SCCs of other sites (32). However, the patient was concerned about the side effects and declined adjuvant chemotherapy, reflecting the ethical challenges of managing rare tumors without consensus guidelines. Among seven reported cases treated with surgery alone, the mean progression-free survival (PFS) was 18.1 months, with a median PFS of 10 months (4–48 months) (**Table 2**). Six cases indicated that the mean overall survival (OS) for surgery alone was 12.8 months, with a median of 3 months (0.8–60 months). Notably, three patients with advanced presentations, including small intestinal perforation, hepatic metastasis, or concurrent malignancies, succumbed within one month postoperatively, underscoring the prognostic impact of tumor burden and comorbidities.

**Table 2:** Survival outcomes under different treatment modalities.

Treatment modality	Median OS (months)	Median PFS (months)
Surgery alone (n=12)	12.8	18.1
Surgery + chemo (n=4)	–	31
Chemo alone (n=1)	1	–
Chemo + Radio (n=3)	18.3	–

Chemo, Chemotherapy; Radio, Radiotherpy.

Adjuvant platinum-based chemotherapy, such as carboplatin/paclitaxel or capecitabine, has shown promise in extending survival. One patient achieved 55 months of OS following carboplatin/paclitaxel therapy (32), while two other patients treated with oxaliplatin combined with capecitabine or 5-fluorouracil/cisplatin and remained recurrence-free at the time of reporting (41, 45). However, outcomes might be stage dependent. Patients with stage II receiving adjuvant chemotherapy demonstrated a PFS of 55 months versus 6 months for surgery alone (32, 37). However, a patient with stage IIIB disease (T4N2M0) survived only one month on chemotherapy alone (34), suggesting the need for performance status-driven dosing. Overall, patients receiving adjuvant chemotherapy had significantly prolonged survival (31 vs. 18.1 months, surgery alone), though small sample sizes limit statistical power.

The role of radiotherapy remains limited. Radiotherapy is a cornerstone treatment for SCC of the head and neck, lung cancer, and rectal cancer (46). However, SCC-SI appears radioresistant, akin to adenocarcinoma (47). Three patients treated with combined chemoradiation achieved a mean OS of 18.3 months, with a median of 17 months, though the lack of stage-specific data in these cases precludes a definitive conclusion (47). Poor outcomes may reflect intrinsic radioresistance or confounding by advanced disease, highlighting the need for cautious interpretation.

Based on aggregated evidence, a stage-guided approach is proposed. For patients with localized disease (Stage I/II), curative resection with negative margins is recommended. For patients with regional lymph node metastasis or a large tumor burden (Stage III), surgery followed by platinum-based adjuvant chemotherapy with dose adjustments for frailty is recommended. For patients with multiple metastases or those who are too frail for surgery, systemic chemotherapy can be administered for palliation. Radiotherapy should be reserved for symptomatic relief in select cases. These recommendations must be contextualized within multidisciplinary frameworks that balance therapeutic intent, patient autonomy, and prognostic realism, particularly give the absence of consensus guidelines for this rare malignancy.

## 4 Conclusion

We report a rare case of the first ileal SCC to a 40-year history of post-surgical fistulas and chronic inflammation, which supports the hypothesis that prolonged mucosal injury may drive squamous metaplasia and malignant transformation. Additionally, we summarize its clinical characteristics and pathogenesis by reviewing reported cases. We emphasize the role of inflammation in the occurrence of SCC and propose that trauma and surgical procedures resulting in the retention and implantation of squamous epithelial cells could be one of the underlying pathogeneses. Determining the best treatment for SCC-SI remains challenging due to its uncommon occurrence.

Currently, there are no reports on the molecular mechanisms for SCC-SI. Only a few studies have reported on and explored the molecular mechanisms of SCC in other locations, such as the rectum, anal canal, and pelvic cavity (48–50). The poor prognosis of SCC-SI underscores the necessity for more research to explore its pathogenesis, thereby facilitating better prevention and treatment. Moreover, prospective studies should explore molecular profiling and immunotherapy in SCC-SI, given their efficacy in SCCs of other sites.

## Data Availability

The original contributions presented in the study are included in the article/**Supplementary Material**. Further inquiries can be directed to the corresponding authors.

## Supplementary material

Supplementary Figure 1 Treatment timeline for this patient.

Supplementary Figure 2 Immunohistochemical staining shows negative expression of CDX2. The left panel is at 40-fold magnification, while the right panel is at 100-fold magnification.
