# Supplementary figures and images for: Squamous cell carcinoma of the small intestine: a case report and review of literature
Source: Front Oncol. 2025 Apr 30;15:1550917. doi: 10.3389/fonc.2025.1550917 (PMC12074906; doi:10.3389/fonc.2025.1550917)

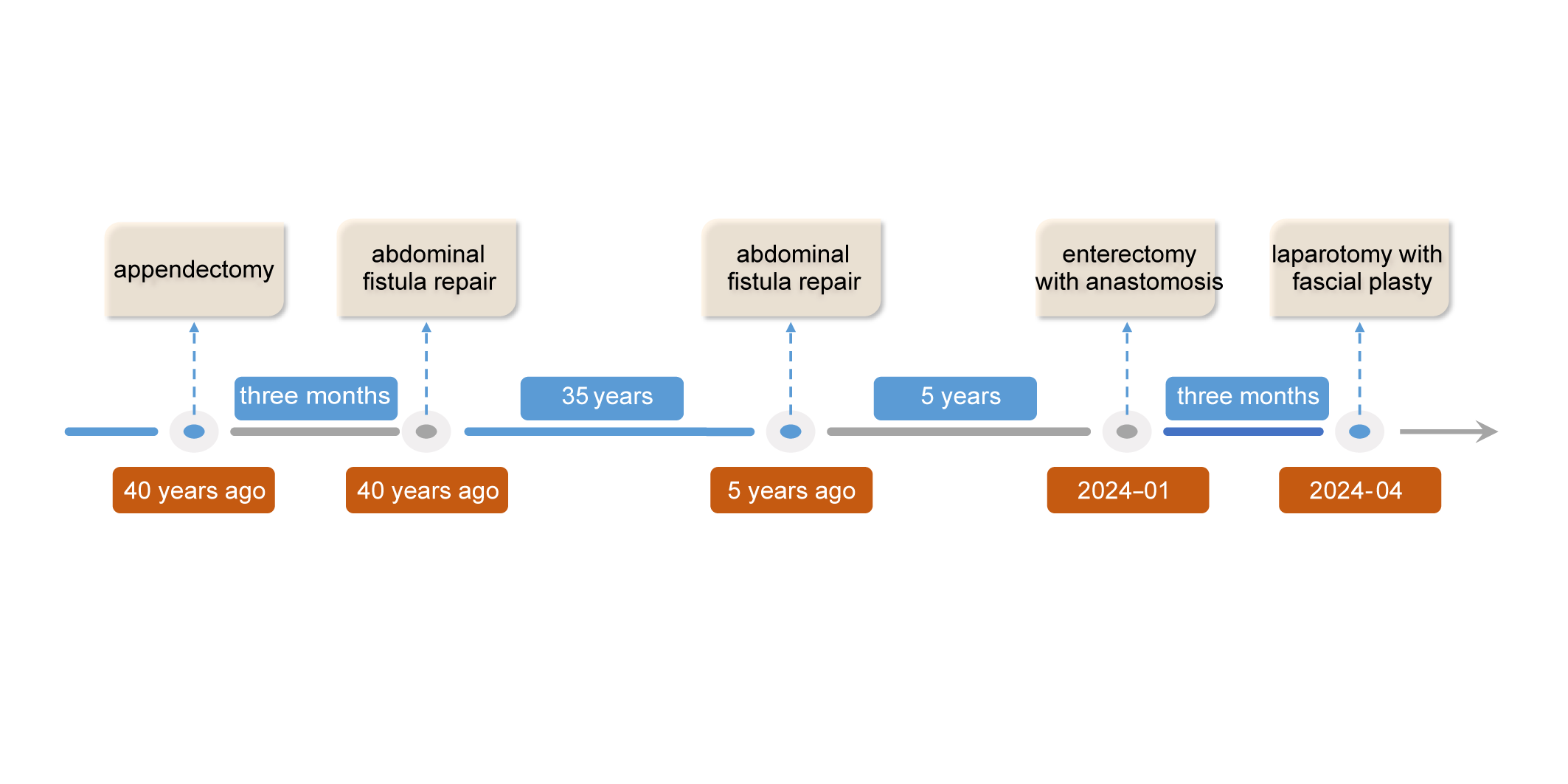

Supplement: Supplementary Figure 1 — Treatment timeline for this patient. [file Image1.tif]

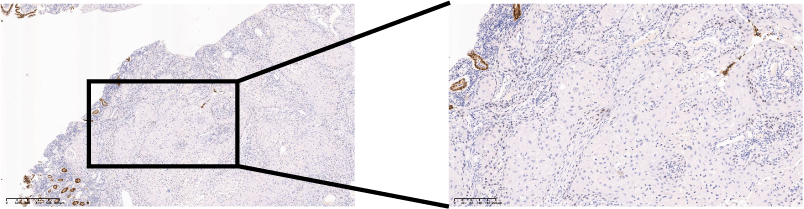

Supplement: Supplementary Figure 2 — Immunohistochemical staining shows negative expression of CDX2. The left panel is at 40-fold magnification, while the right panel is at 100-fold magnification. [file Image2.tif]
